# Morphological Analyses of Effects of Endodontic Irrigant Solutions Using a Root Canal Model and an Immersion Model

**DOI:** 10.1155/2023/3938522

**Published:** 2023-07-26

**Authors:** Yuzo Kawanishi, Hazuki Maezono, Tsuyoshi Shimaoka, Takumi Kitatani, Katsuaki Naito, Maki Sotozono, Kittipit Klanliang, Yusuke Takahashi, Mikako Hayashi

**Affiliations:** ^1^Department of Restorative Dentistry and Endodontology, Osaka University Graduate School of Dentistry, 1-8 Yamada-Oka Suita, Osaka 565-0871, Japan; ^2^Division of Cariology, Operative Dentistry and Endodontics, Department of Oral Health Science, Niigata University Graduate School of Medical and Dental Sciences, 2-5274 Gakkocho-Dori Chuo-Ku, Niigata 951-8514, Japan; ^3^Division of Endodontics, Department of Restorative Dentistry and Periodontology, Faculty of Dentistry, Chiang Mai University, Suthep Road Tambon Suthep Amphur Mueang Chiang Mai, Chiang Mai 50200, Thailand

## Abstract

**Objective:**

This study aimed to compare an experimental model simulating clinical root canal irrigation (root canal model) with a conventional experimental model immersing dentin sample to irrigants (immersion model) to evaluate removal of the smear layer and decalcification of the root canal dentin using sodium hypochlorite (NaOCl) and two different concentrations of ethylenediaminetetraacetic acid (EDTA) solution.

**Materials and Methods:**

Forty-five single-rooted extracted human teeth were prepared using a Ni–Ti rotary file. EDTA, NaOCl, and citric acid were used in the root canal models and the immersion models. After the irrigation protocol, root canal surfaces were observed under scanning electron microscopy. Residual smear and decalcification of the root canal dentin were evaluated objectively by measuring the percentage of the area occupied by visible dentin tubules, the number of visible dentin tubules, and the mean area of a visible single dentin tubule.

**Results:**

Root canal and immersion models with the same irrigation protocol showed significantly different results for smear residues and decalcification of root canal dentin. In the root canal model, neither different EDTA concentrations nor the order of EDTA and NaOCl applications significantly impacted smear residues or decalcification of root canal dentin. Furthermore, no erosion of the root canal dentin surface was observed in any experimental groups in the root canal model using EDTA and NaOCl compared to intact dentin.

**Conclusions:**

Experimental design affected results for residual smear layer and decalcification of root canal dentin. The order of EDTA and NaOCl use and the concentration of EDTA did not affect results. EDTA and NaOCl irrigation did not cause erosion in the root canal model in this study.

## 1. Introduction

A smear layer is an amorphous film formed during mechanical root canal preparation [1–3]. This layer adheres to the root canal wall and harbors microorganisms [1, 3], prevents or delays diffusion of irrigants and medicaments into dentinal tubules [4], and inhibits penetration of the sealer into dentinal tubules [5]. As a result, the sealing ability of the root canal is compromised, increasing the risk of reinfection [6, 7].

Chelating agents have been suggested for removal of the smear layer, as well as for decalcification of the root canal dentin [8, 9], and previous studies have reported alternating irrigation with the calcium chelator ethylenediaminetetraacetic acid (EDTA) and the organic tissue solvent sodium hypochlorite (NaOCl) as an effective method of removing the smear layer formed on the dentin surface of the root canal [4, 6, 10]. On the other hand, prolonged activity of EDTA solution and the order of applying irrigants have been suggested to potentially cause erosion of the root canal dentin, which may compromise dentin strength [11–13]. However, in many reports on morphological evaluations of smear layer removal and decalcification of the root canal dentin surface using various cleaning solutions, dentin blocks were immersed in the solutions [11, 13, 14], and few reports have used models that maintain the morphology of the root canal system. In addition, although various concentrations of EDTA products are now commercially available, few reports appear to have described quantitative morphological analyses of the abilities to remove the smear layer and decalcify root canal dentin using EDTA at different concentrations and also in different orders [15].

This study aimed to morphologically investigate the ability to remove the smear layer and decalcify root canal dentin using EDTA and NaOCl solutions in an experimental model simulating clinical root canal irrigation. The results were compared to those of a conventional experimental model immersing dentin samples. The effects of different concentrations of EDTA solution were also investigated.

## 2. Materials and Methods

This study was approved by the Ethics Committee of Osaka University Graduate School of Dentistry (permit no. R1-E44).

### 2.1. Sample Preparation

**Figure 1:**
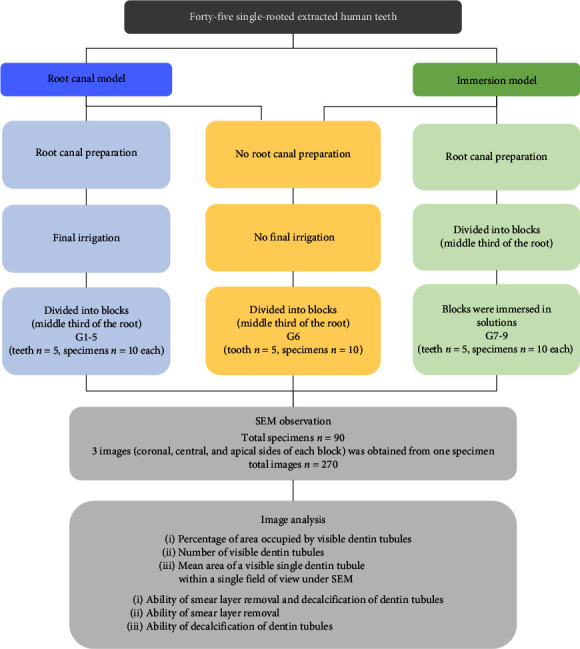
Experimental protocol.

Forty-five single-rooted human teeth extracted for periodontal and orthodontic reasons, including maxillary premolars, mandibular incisors, and mandibular premolars, were stored in saline until use. These 45 teeth were from individuals in their 20s to 70s with sound crowns and roots and no history of caries, restorative treatments, or root canal treatments. The experimental protocol is shown in Figure 1. The tooth crown was removed and root length was set at 12 mm. The working length was determined by subtracting 1 mm from the length of the apical foramen, as measured by a size #10 K-file (Dentsply Sirona, Charlotte, NC) and the root was implanted in silicone putty (Exafine putty type; GC, Tokyo, Japan). The glide path was prepared using a #15 K file (Dentsply Sirona). Ni–Ti rotary files (Race; FKG Dentaire, La Chauxde-Fonds, Switzerland) and an endodontic motor (MiniENDO; FKG Dentaire) were used for root canal preparation following the protocol recommended by the manufacturer. After pre-enlargement with a #35.08, the root canal was prepared using a crown-down technique in the order of #30.06, #30.04, #25.04, and #25.02 until the file reached the working length. The root canal was then enlarged until #30.06. Just before using each file, 0.5 ml of NaOCl (Neo cleaner; Neo Dental Chemical Products, Tokyo, Japan) was utilized for irrigation, and a maximum of eight Ni–Ti files were used. When less than eight files were used for root canal preparation, extra NaOCl was applied to adjust the total amount of NaOCl to 4 ml in order to unify the amount of NaOCl used for irrigation. Irrigant was delivered via a 25-G syringe (root canal syringe; Neo Dental Chemical Products). After preparation, the inside of the root canal was rinsed with sufficient amount of distilled water, and the root canal was dried using #30 paper points (VDW, Munich, Germany).

### 2.2. Root Canal Model

Final irrigation was performed using 2.5% NaOCl, 3% EDTA (pH = 9.5) (Smear Clean; Nippon Shika Yakuhin, Shimonoseki, Japan), 18% EDTA (pH = 12) (Ultradent, South Jordan, UT), and 10% citric acid (CA). Experimental groups using the root canal model were set as shown in Table 1.

Each solution was dispensed into the canal at 0.5 ml per 10 s and used at room temperature. Passive ultrasonic irrigation (PUI) was performed using an ultrasonic device (Suprasson P-MAX; Acteon Satelec, Merignac, France) with a #25 file-shaped instrument (AM file; Acteon Satelec), inserting the tip of the ultrasonic instrument to the working length 2 mm in P3 mode, as recommended by the manufacturer. The duration of irrigation with EDTA was set to 1 min and the duration of NaOCl with PUI was set to 30 s, based on a previous report [16]. After final irrigation, samples were dried with a #30 paper point and washed with distilled water. A diamond disc (Horico diamond disc; Hopf, Ringleb, Berlin, Germany) was used to make a groove in the buccolingual direction of the tooth axis, and a razor blade was used to divide the groove into two sections. Each piece was then divided into three sections of 4 mm each using the same method, and the central portion of the root was used for observation.

### 2.3. Immersion Model

The experimental protocol was performed in accordance with the method of Qian et al. [11]. In brief, after root canal preparation, the tooth root was divided into blocks as the root canal model, and then the blocks were immersed in 2.5% NaOCl, 18% EDTA, and 10% CA in centrifuge tubes. Tubes were placed in an ultrasonic cleaner device (UT306; Sharp, Sakai, Japan) for ultrasonic irrigation with NaOCl. Blocks were rinsed with distilled water after immersion and used for observation. Experimental groups using the immersion model were set as shown in Table 2.

### 2.4. Scanning Electron Microscopy (SEM)

Specimens (*n* = 90) were dehydrated and coated with gold sputter [11]. Using a scanning electron microscope (JSM-6390LV; JEOL, Tokyo, Japan), a total of 270 images of the middle-third root canal dentin at three locations (coronal, central, and apical sides of each block) were randomly obtained at 20 kV and ×1,300 magnification by an observer blinded to the sample preparation and experimental groups.

### 2.5. Image Analysis

**Figure 2:**
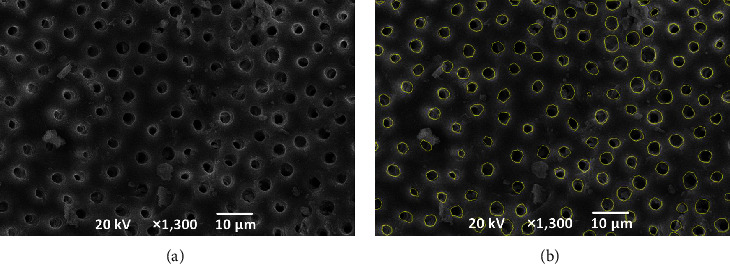
Example of an SEM image (a) and example of an SEM image tracing dentin tubules not covered by smear using imageJ (b).

Images obtained by SEM were transferred to a computer and examined. ImageJ (version 1.53k; National Institutes of Health, Bethesda, MD) was used to trace visible dentin tubules not covered by the smear (Figure 2), and the percentage of the area occupied by visible dentin tubules was calculated by comparing the total area of visible dentin tubules to the total area within a single field of view under SEM. The number of traced visible dentin tubules was measured and the number of visible dentin tubules was calculated. Finally, the mean area of a visible single dentin tubule was obtained by dividing the total area of dentin tubules by the number of dentin tubules in the same field of view. These measurements were taken by another observer also blinded to the sample preparation and experimental groups. We defined erosion of the root canal dentin as a significantly larger mean area of a visible single dentin tubule compared to G6 (no preparation, no irrigation).

### 2.6. Statistical Analyses

Results for the percentage of visible dentin tubules, number of visible dentin tubules and the mean area of a visible single dentin tubule are expressed as mean ± standard error of the mean.

Statistical analyses were performed using IBM SPSS Statistics version 22.0 (IBM Corp, Armonk, NY). The Shapiro–Wilk test was used to test for normality, the Levene test was used to assess homoscedasticity, the *t*-test was used for parametric comparisons between two groups, and the Mann–Whitney *U* test was used for nonparametric comparisons.

Since all comparisons between multiple groups were nonparametric, Kruskal–Wallis and Bonferroni-corrected Dunn tests were performed. The significance level was set at 5%.

## 3. Results

**Figure 3:**
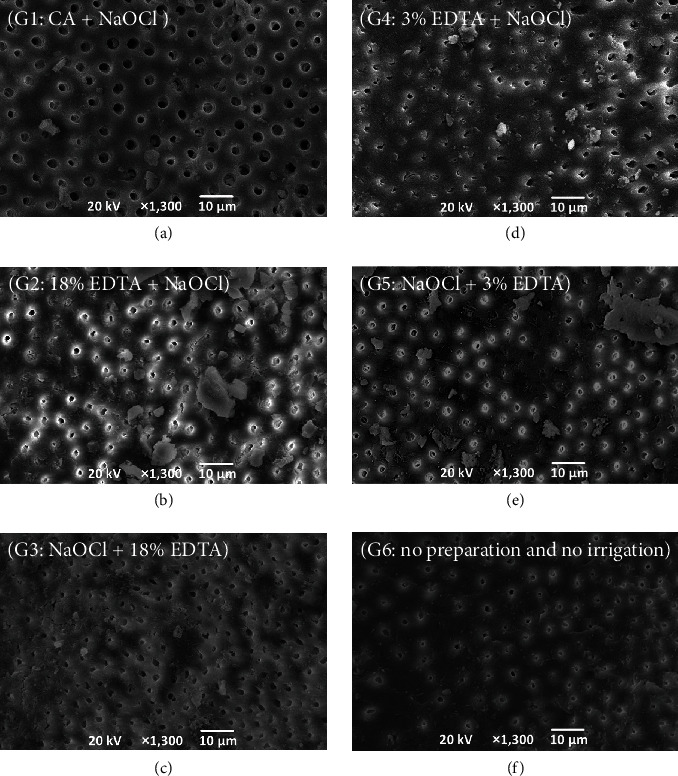
Representative scanning electron microscopy images of root canal dentin in the root canal models after irrigation (×1,300 magnification): (a) G1 (CA/PUI + NaOCl/PUI), (b) G2 (18% EDTA + NaOCl/PUI), (c) G3 (NaOCl/PUI + 18% EDTA), (d) G4 (3% EDTA + 2.5% NaOCl/PUI), (e) G5 (NaOCl/PUI + 3% EDTA), and (f) G6 (no preparation and no irrigation).

Figure 3 provides representative images of each group with different irrigation methods in the root canal models. When CA and NaOCl were used (Figure 3(a)), few smear layers were observed but peritubular dentin was eroded. In groups using EDTA and NaOCl (Figure 3(b)–3(e)), a small amount of smear layer was present, but no obvious erosion of dentin tubules was observed. Percentage of the area occupied by visible dentin tubules under SEM was significantly higher in G1 compared to G2–5 (*P* < 0.05), and did not differ among G2–5 (*P* ≥ 0.05) (Table 3). No significant difference in the number of visible dentin tubules was observed between any groups (*P* ≥ 0.05) (Table 4). Mean area of a visible single dentin tubule was significantly wider in G1 than in G2–6 (*P* < 0.05), and did not differ among G2–6 (*P* ≥ 0.05) (Table 5).

**Figure 4:**
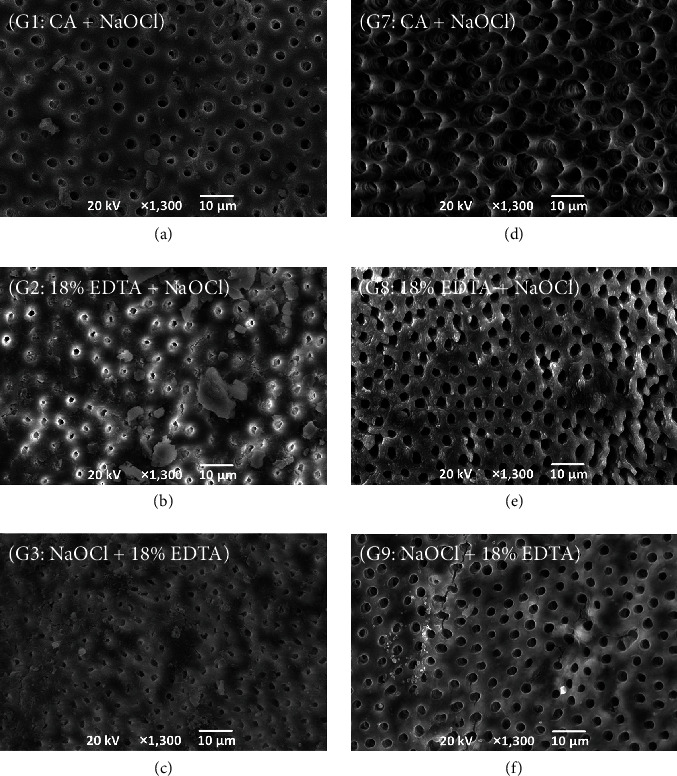
Representative scanning electron microscopy images of root canal dentin comparing root canal models: (a–c) and immersion models; (d–f) (×1,300 magnification); (a) G1 (root canal model: CA/PUI + NaOCl/PUI), (b) G2 (root canal model: 18% EDTA + NaOCl/PUI), (c) G3 (root canal model: NaOCl/PUI + 18% EDTA), (d) G7 (immersion model: CA/ultrasonic + NaOCl/ultrasonic), (e) G8 (immersion model: 18% EDTA + NaOCl/ultrasonic), and (f) G9 (immersion model: NaOCl/ultrasonic + 18% EDTA).

Representative images for comparison between root canal models and immersion models were obtained by SEM (Figure 4). Although no smear layer was observed in the immersion models (Figure 4(d)–4(f), dentin tubules exhibited clear erosion compared to those in root canal models. Immersion models showed a significantly higher percentage of visible dentin tubules and higher number of visible dentin tubules than root canal models (*P* < 0.05 each) (Tables 6 and 7). In addition, mean area of a visible single dentin tubule was significantly larger for immersion models (*P* < 0.05) (Table 8).

## 4. Discussion

The present study compared immersion and root canal models in terms of decalcification of root canal dentin and removal of the smear layer. We also examined the effects of the order of NaOCl and EDTA use and EDTA concentration on decalcification of root canal dentin and smear layer removal in the root canal model.

To the best of our knowledge, no reported experiments have quantitatively evaluated the percentage of the area occupied by visible dentin tubules, number of dentin tubules, and mean area of a visible single dentin tubule using NaOCl and EDTA in root canal models. We therefore considered that calculation of the appropriate sample size for this study was difficult. In the present study, we used a sample size equivalent to previous reports that evaluated removal of the smear layer and demineralization of the root canal dentin morphologically [17].

Various reports have evaluated scores for smear layer removal and decalcification of root canal dentin [18, 19]. However, score evaluations have been ambiguous and have varied among evaluators. This study therefore used a more objective evaluation method. The percentage of the area occupied by visible dentin tubules, number of visible dentin tubules, and mean area of a visible single dentin tubule represent objective data obtained by SEM image analysis. The percentage of area occupied by visible dentin tubules indicate the ability of smear layer removal and decalcification of root canal dentin. The number of visible dentin tubules can indicate the ability of smear layer removal. The mean area of a visible single dentin tubule can indicate decalcification of root canal dentin.

In a previous report on immersion systems [11], the group using 10% CA + 5.25% NaOCl showed more dentin demineralization than the group using EDTA and NaOCl. We therefore used 10% CA + 2.5% NaOCl as a positive control group. The same protocol for 18% EDTA, NaOCl, and CA was used in both the root canal model (G1, G2, and G3) and the immersion model (G7, G8, and G9). The results showed significant differences in the ability to remove the smear layer and decalcify root canal dentin between the two models. Previous studies have reported that irrigation using EDTA followed by NaOCl could cause erosion [11, 12]. However, those studies were performed using morphological evaluations and elemental analysis in an immersion model, but not in the root canal model, and thus may not be in-line with actual clinical practice. In the immersion model, too much irrigation caused excessive reactions, representing a point of difference from clinical practice. In our study, no differences in smear removal or decalcification were seen with differences in the order of EDTA and NaOCl use in the root canal model. Although this differs from a previous study [11], which also suggests that the experimental model may have influenced the results. Since the results of the immersion system differed from those of the root canal system that imitated the clinical situation, use of the root canal system was considered appropriate when evaluating the effect of root canal irrigating solution on the root canal dentin. In the root canal model, no significant differences were observed when the order of use of EDTA and NaOCl or the concentration of EDTA was changed, suggesting that differences in the order of use of EDTA and NaOCl or the EDTA concentration used in this experiment had no effect on the ability to remove the smear layer or decalcification of the root canal dentin. In addition, no difference was observed between G2–5 and G6 in terms of mean area of a visible single dentin tubule, suggesting that under the conditions of this experiment, no erosion occurred when NaOCl and EDTA were used together for final irrigation. This experiment had some limitations, as no classifications were made with respect to the age of teeth used in this study, and the specimens examined were only the middle third of the root. Further study of these issues is therefore needed.

This study used two concentrations of EDTA, 3% and 18%. Previous studies measuring free phosphorus concentrations have reported that higher concentrations of EDTA [20] and closer-to-neutral pH are more likely to result in decalcification [19, 20]. In the present study, two different concentrations of EDTA products did not show any significant differences in residual smear layer or decalcification of root canal dentin (Figure 4), and these results are similar to a previous study using morphological assessment [15]. The 3% EDTA (pH = 9.5) used in this study was closer to neutral, and thus might have had the same chelating ability as the higher concentration 18% EDTA (pH = 12). As further investigations, the concentrations and pH of EDTA should be controlled to clarify the precise effects on the dentin surface after irrigation.

NaOCl is generally used at concentrations ranging from 0.5% to 8.25% for root canal treatment [21]. This chemical is capable of completely eliminating *Enterococcus faecalis* and dissolving dental pulp tissue when used at high concentration [21, 22]. However, high concentrations of NaOCl are cytotoxic and may cause side effects [23]. In addition, alternating use of NaOCl and EDTA reportedly decreases surface strain of root canal dentin [24]. Low concentrations of NaOCl are less cytotoxic, but have reduced antibacterial activity and organic tissue-solubilizing ability [25]. One report found that concentration differences in NaOCl did not affect the ability to remove the smear layer [26]. We usually use 2.5% NaOCl in root canal treatments, based on the antimicrobial effects, tissue solubility, and cytotoxicity, so we decided to use 2.5% NaOCl in this study. A systematic review [27] comparing PUI and syringe irrigation found no difference in the healing of apical periodontitis after initial treatment. PUI was effective in removing pulp tissue remnants and hard tissue debris in vitro, although conflicting results regarding antimicrobial effect have been reported [27].

In the present study, NaOCl was used in combination with PUI as usually performed in clinical situations, and EDTA was used statically without PUI following product instructions. Although the EDTA usage time recommended by the manufacturer is 2 min for the 3% solution and ≤1 min for the 18% solution, we set the usage time as 1 min, as recommended in a previous study [13] to compare the two EDTA solutions. The position of the PUI tip is often set at working length minus 1–2 mm in previous studies [28, 29], and we set the tip at working length minus 2 mm in this study, as we usually use.

The experimental model design affected the results for residual smear layer and decalcification of root canal dentin, whereas the order of EDTA and NaOCl use and the concentration of EDTA did not. EDTA and NaOCl irrigation did not cause erosion of root canal dentin in the root canal model.

## Figures and Tables

**Table 1:** Irrigation protocol of root canal models.

Group	Irrigation methods
G1 (*n* = 5)	10% CA (PUI) for 60 s × 5 + 2.5% NaOCl (PUI) for 30 s
G2 (*n* = 5)	18% EDTA for 60 s + 2.5% NaOCl (PUI) for 30 s
G3 (*n* = 5)	2.5% NaOCl (PUI) for 30 s + 18% EDTA for 60 s
G4 (*n* = 5)	3% EDTA for 60 s + 2.5% NaOCl (PUI) for 30 s
G5 (*n* = 5)	2.5% NaOCl (PUI) for 30 s + 3% EDTA for 60 s
G6 (*n* = 5)	No preparation and no irrigation

**Table 2:** Irrigation protocol of immersion models.

Group	Irrigation methods
G6 (*n* = 5)	No preparation and no irrigation
G7 (*n* = 5)	10% CA (ultrasonic) for 60 s × 5 + 2.5% NaOCl (ultrasonic) for 30 s
G8 (*n* = 5)	18% EDTA for 60 s + 2.5% NaOCl (ultrasonic) for 30 s
G9 (*n* = 5)	2.5% NaOCl (ultrasonic) for 30 s + 18% EDTA for 60 s

**Table 3:** Percentage of the area occupied by visible dentin tubules (mean ± SE) among various irrigation sequences in root canal models.

Groups	G1	G2	G3	G4	G5	G6
Irrigation methods	CA + NaOCl	18% EDTA + NaOCl	NaOCl + 18% EDTA	3% EDTA + NaOCl	NaOCl + 3% EDTA	No preparation and no irrigation
Percentage (%) ± SE	16.64^a^ ± 3.62	1.96^b^ ± 0.32	2.36^b^ ± 0.37	2.36^b^ ± 0.47	1.96^b^ ± 0.43	N/A

Different letters represent significant differences among groups (*P* < 0.05). N/A: not applicable.

**Table 4:** Number of visible dentin tubules (mean ± SE) among various irrigation sequences in root canal models.

Groups	G1	G2	G3	G4	G5	G6
Irrigation methods	CA + NaOCl	18% EDTA + NaOCl	NaOCl + 18% EDTA	3% EDTA + NaOCl	NaOCl + 3% EDTA	No preparation and no irrigation
Number ± SE	135.91 ± 12.87	83.73 ± 9.56	100.93 ± 13.99	104.47 ± 13.02	80.13 ± 10.79	N/A

**Table 5:** Mean area of a visible single dentin tubule (mean ± SE) among various irrigation sequences in root canal models.

Groups	G1	G2	G3	G4	G5	G6
Irrigation methods	CA + NaOCl	18% EDTA + NaOCl	NaOCl + 18% EDTA	3% EDTA + NaOCl	NaOCl + 3% EDTA	No preparation and no irrigation
Mean area (*μ*m^2^) ± SE	9.16^a^ ± 1.55	1.70^b^ ± 0.13	1.85^b^ ± 0.19	2.50^b^ ± 0.37	1.68^b^ ± 0.17	1.89^b^ ± 0.34

Different letters represent significant differences among groups (*P* < 0.05). N/A: not applicable.

**Table 6:** Percentage of the area occupied by visible dentin tubules (Mean ± SE) between root canal models and immersion models using the same irrigation sequences.

Groups	G1	G7	G2	G8	G3	G9
	CA + NaOCl		18% EDTA + NaOCl		NaOCl + 18% EDTA	
Irrigation methods	Root canal	Immersion	Root canal	Immersion	Root canal	Immersion
Percentage (%) ± SE	16.64 ± 3.62	54.84 ± 4.21	1.96 ± 0.32	16.39 ± 1.53	2.36 ± 0.37	11.13 ± 1.48
*P*-value	<0.05		<0.05		<0.05	

**Table 7:** Number of visible dentin tubules (Mean ± SE) between root canal models and immersion models using the same irrigation sequences.

Groups	G1	G7	G2	G8	G3	G9
	CA + NaOCl		18% EDTA + NaOCl		NaOCl + 18% EDTA	
Irrigation methods	Root canal	Immersion	Root canal	Immersion	Root canal	Immersion
Number ± SE	135.91 ± 12.87	177.73 ± 10.08	83.73 ± 9.56	212.67 ± 12.43	100.93 ± 13.99	162.47 ± 14.92
*P*-value	<0.05		<0.05		<0.05	

**Table 8:** Mean area of a visible single dentin tubule (Mean ± SE) between root canal models and immersion models using the same irrigation sequences.

Groups	G1	G7	G2	G8	G3	G9
	CA + NaOCl		18% EDTA + NaOCl		NaOCl + 18% EDTA	
Irrigation methods	Root canal	Immersion	Root canal	Immersion	Root canal	Immersion
Mean area (*μ*m^2^) ± SE	9.16 ± 1.55	25.1 ± 2.75	1.70 ± 0.13	5.83 ± 0.49	1.84 ± 0.19	5.27 ± 0.45
*P*-value	<0.05		<0.05		<0.05	

## Data Availability

Data are available upon request via email to the corresponding author.
